# High-Resolution Genomic Profiling of Chromosomal Abnormalities in Human Stem Cells Using the 135 K StemArray

**DOI:** 10.1155/2012/431534

**Published:** 2012-04-05

**Authors:** Aaron M. Elliott, Kristi A. Hohenstein Elliott, Anja Kammesheidt

**Affiliations:** Ambry Genetics, Departments of Genomic Services and R&D, 15 Argonaut, Aliso Viejo, CA 92656, USA

## Abstract

Culturing stem cells for an extended period of time can lead to acquired chromosomal aberrations. Determining the copy number variant (CNV) profile of stem cell lines is critical since CNVs can have dramatic effects on gene expression and tumorigenic potential. Here, we describe an improved version of our StemArray, a stem-cell-focused comparative genomic hybridization (aCGH) microarray, which contains 135,000 probes and covers over 270 stem cell and cancer related genes at the exon level. We have dramatically increased the median probe spacing throughout the genome in order to obtain a higher resolution genetic profile of the cell lines. To illustrate the importance of using the StemArray, we describe a karyotypically normal iPSC line in which we detected acquired chromosomal variations that could affect the cellular phenotype of the cells. Identifying adaptive chromosomal aberrations in stem cell lines is essential if they are to be used in regenerative medicine.

## 1. Introduction

Several studies have demonstrated that human embryonic and induced pluripotent stem cells (ESCs, iPSCs) acquire genomic abnormalities during prolonged culture [1–3]. These chromosomal aberrations can have dramatic effects on the survival, proliferative ability, and differentiation potential of the cells, which can result in unreliable experimental results and jeopardize their potential use in regenerative medicine. The most common method used by stem cell researchers to monitor the genomic stability of the cell lines is G-banded karyotype analysis. However, this method can only detect large variations over 5 megabases (Mb), and therefore, the majority of smaller chromosomal changes are missed. Recently, numerous groups have been employing other methods for stem cell characterization, including gene expression profiling and array-based comparative genomic hybridization (aCGH) microarray analysis [4–6]. Although gene expression profiling is beneficial to illustrate the true transcriptional state of the cells, the resolution of this technique is over 10 Mb in size [4]. aCGH is a technique which can detect unbalanced structural abnormalities at a typical resolution under 100 Kb. Studies using aCGH microarrays to detect copy number variations in stem cells have identified numerous subkaryotypic alterations acquired during cultural adaptation [2, 6]. However, the arrays used in these studies were nontargeted whole genome tiling arrays, which generally have low coverage of single genes and are relatively expensive for routine analysis. 

We previously developed a stem-cell-targeted aCGH microarray which contains 44 K probes with increased probe coverage in targeted regions [7]. Here, we describe an updated and improved version of the StemArray that is currently used by a wide variety of stem cell laboratories to characterize the genomic integrity of their stem cell lines. The array contains 135 K probes to cover the entire genome at an average resolution of 15 Kb. In addition, the custom-targeted microarray has exon level resolution in over 270 stem cell and cancer-related genes. The use of the 12 × 135 K array platform, which allows 12 samples to be run per slide, significantly reduces the costs of the array and makes it competitive in pricing with karyotype analysis. 

## 2. Materials and Methods

### 2.1. iPSC Line Culture

iPSC lines used in the study were generated from fibroblasts using standard retroviral transduction of *OCT4*, *SOX2*, *KLF4,* and *c-MYC*. Resulting iPSC lines were cultured on Matrigel (BD Biosciences) substrates in conditions described previously [7]. Genomic DNA was isolated using the Puregene DNA purification Kit (Qiagen) and the quality determined using an ND-1000 spectrophotometer (NanoDrop).

### 2.2. aCGH

The stem-cell-focused microarray was developed by Ambry Genetics (Aliso Viejo, CA) using Roche NimbleGen probe sets. The microarray contains 135,000 probes annotated against the human genome assembly build 37 (UCSC hg 19). Probe density was increased in over 270 stem cell and cancer-associated genes, with an average of in these regions (gene list available upon request). The remaining probes were tiled throughout the genomic backbone at a median probe spacing of 15 Kb. Following validation runs, only those probes with optimal performance were selected for the final array design. aCGH was performed according to the Roche NimbleGen protocol (V.8.0). Briefly, 500 ng of human stem cell DNA and 500 ng of pooled sex-matched reference DNA (Promega) were heat denatured at 98°C for 10 minutes and then labeled with Cyanine 3 Random Nonamers and Cyanine 5 Random Nonamers by Exo-Klenow fragment. The labeled DNA was then purified by isopropanol precipitation and the labeling efficiency determined using an ND-1000 spectrophotometer. Based on the concentration, 20 *μ*g of the labeled sample and reference DNA along with 2X hybridization buffer, hybridization component A, and alignment oligo were added together and placed on the 135 K StemArray (Ambry Genetics). Microarrays were hybridized on the Maui Hybridization System (Roche NimbleGen) at 42°C for 72 hours. Slides were washed according to the protocol and scanned at 2 *μ*M resolution on a NimbleGen MS200 high resolution scanner.

### 2.3. Data Analysis

Data was extracted and normalized using NimbleScan 2.6 software package (Roche NimbleGen). For aberration calling, normalized data sets were imported into Nexus Copy Number version 6.0 (BioDiscovery). To correct for GC content, a noise reducing systematic correction file was developed based on the genomic locations of the probes in the design. Aberrant regions were determined using the FASST2 segmentation algorithm with a significance threshold of 1.0E-6. The aberration filter was selected with the following parameters: minimum number of probes in the region 4, minimum absolute average log_2 _ratio for one copy amplification was  .3 and for a heterozygous deletion −.3, and a mean log_2_ ratio ≥1.0 represents a high copy gain and ≤1.1 a homozygous copy loss.

## 3. Results and Discussion

**Figure 1:**
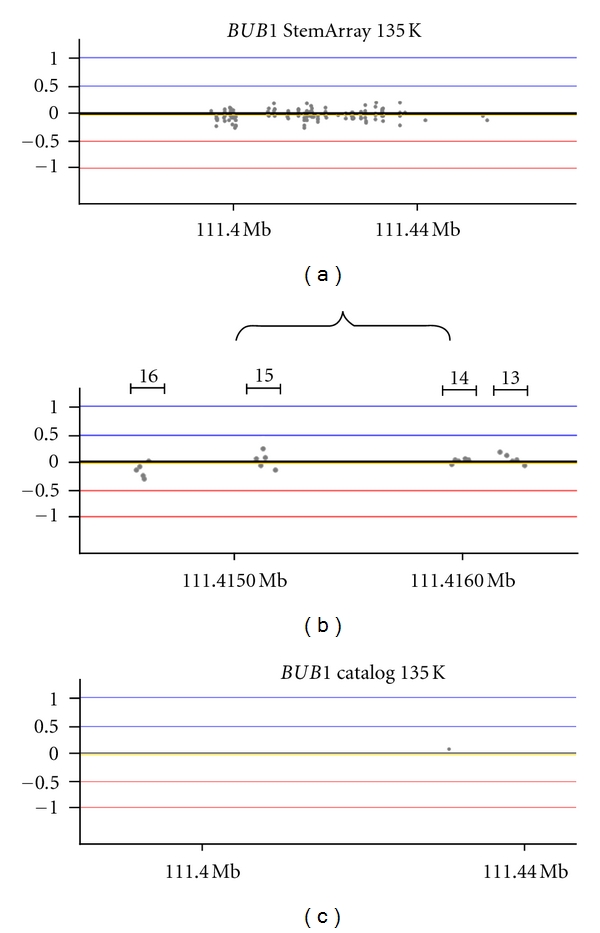
The 135 K StemArray has increased probe coverage in stem cell and cancer-associated genes. (a) Stemness gene *BUB1 *contains 141 total probes resulting in single exon resolution. (b) Exons are covered with 5 probes/exon enabling the detection of a single exon deletion or amplification. (c) In comparison, the standard NimbleGen catalog 135 K array only contains a single probe in the *BUB1 *gene and therefore does not have the resolution to detect a variation in this gene.

In an effort to identify smaller intragenic variations in genes important for stem cell maintenance, we have made significant improvements to the StemArray design published previously [7]. The probe content has increased from 44 K probes to 135 K probes, resulting in an overall increase in backbone resolution from 43 Kb to 15 Kb, respectively. The updated design also includes on average of 5 probes per exon in over 270 stem cell and cancer-related genes, enabling single exon resolution in these functionally important regions. For example, the 28 exons of the kinase BUB1, a key “stemness” gene essential for maintaining genomic stability, are covered at the exon level by 141 probes (Figures 1(a) and 1(b)) [8, 9]. In contrast, the standard catalog 135 K nontargeted array contains only one probe in the *BUB1* gene (Figure 1(c)).

**Figure 2:**
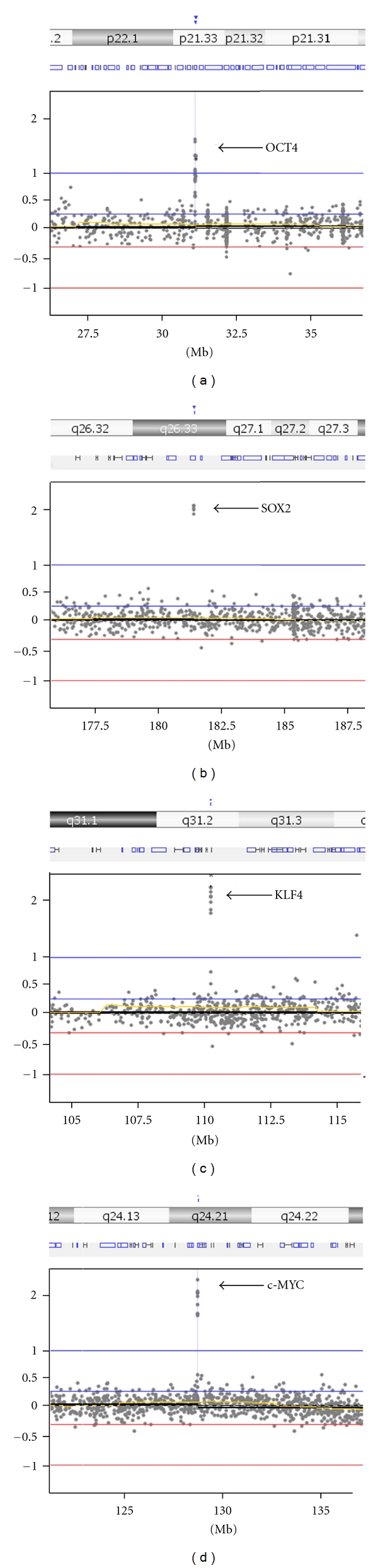
iPSC lines are typically derived by transforming fibroblast cells with retroviral vectors containing *OCT4*, *SOX2*, *KLF4,* and *c-MYC*. These reprogramming genes provide excellent positive controls for the microarray as multiple duplications for each transgene should be observed. The updated 135 K StemArray can detect multiple copy integrations of the iPSC transforming factors (a) *OCT4*, (b) *SOX2*, (c) *KLF4*, and (d) *c-MYC*.

iPSC lines are generally derived by transforming fibroblast cells with retroviral vectors containing *OCT4*, *SOX2*, *KLF4,* and *c-MYC *[10]. These genes provide good positive controls for iPSC aCGH data since multiple copies of these transgenes integrate into the DNA. Using our previous 44 K design, we were not able to detect transgene integration of *OCT4* due to lack of quality probes in the exons. However, with the new 135 K design, we are able to identify high copy amplifications of all four pluripotency genes (Figures 2(a)–2(d)).

**Figure 3:**
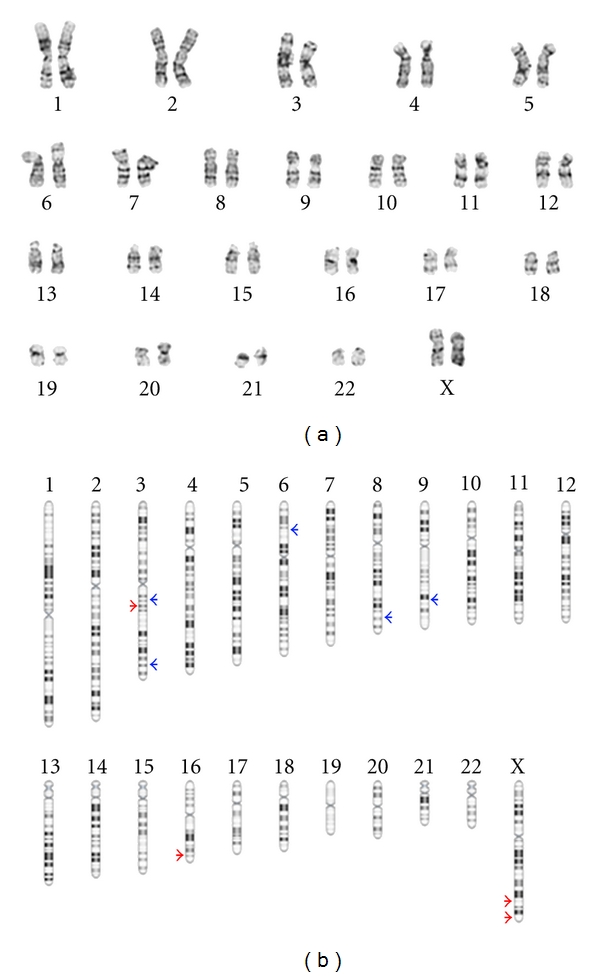
Genomic stability profiling of a human iPSC line by karyotype analysis and the StemArray. (a) The majority of stem cell researchers still characterize their cells by G-banding metaphase karyotyping which has a resolution of only 5 Mb. Testing our iPSC line with this method did not detect any aberrations. (b) aCGH with the custom 135 K microarray identified 4 deletions and 5 amplifications in the iPSC sample ranging in size from 1.5 Kb to 595 Kb.

To illustrate the importance of characterizing stem cell lines with a stem-cell-focused microarray, we monitored the genomic stability of a late passage iPSC line by both G-banded karyotyping and the custom focused 135 K StemArray. Karyotype analysis revealed no aberrations in this iPSC line (Figure 3(a)). Following this result, most stem cell researchers would consider this cell line normal and suitable for further research. However, high-resolution aCGH analysis revealed 9 subkaryotypic variations in the iPSC line ranging in size from 1.5 Kb to 595 Kb (Figure 3(b)).

When conducting aCGH on an iPSC line, it is recommended to first determine the genomic profile of the parental fibroblast line from which the cells were derived. Since all individuals genomic DNA contain copy number variations, performing this initial test will allow one to separate the chromosomal variations acquired during culture from those inherent to the parental fibroblasts. Moreover, it is not uncommon for fibroblast lines to acquire genomic alterations in culture similar to stem cells. Performing aCGH on these cells before one derives iPSC lines is good practice as one would not want to waste time and money developing stem cell lines from fibroblasts which already contain detrimental aberrations. By doing this, we could classify 7 of the identified variations as derived from either the integration of the 4 transgenes, or as copy number variations present in the parental fibroblast population. Therefore, the remaining 2 aberrations had been acquired by the iPSC line during reprogramming or prolonged culture.

**Figure 4:**
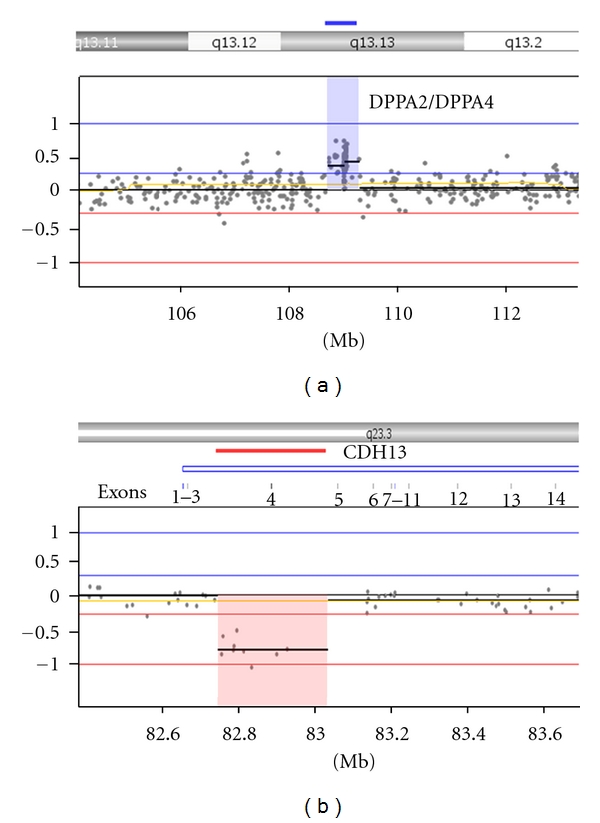
The stem-cell-targeted 135 K StemArray can detect causative aberrations in iPSC lines known to influence cell survival and proliferation. Detection of two acquired chromosomal abnormalities in stem-cell-associated and cancer-related genes in an iPSC line. (a) A 595 Kb amplification spanning the stem-cell-related DPPA2 and DPPA4 genes, and (b) a 285 Kb deletion covering exons 4-5 of the cancer associated CDH13 gene.

The 595 Kb amplification at 3q13.13 contains 8 genes including the stem-cell-associated *DPPA2* and *DPPA4 *genes (Figure 4(a)). Several studies have identified these tightly linked genes as specific markers for pluripotent cells [11–13]. The function of *DPPA2* and *DPPA4* in stem cells has been controversial. Madan et al. [14] created *DPPA2*/*DPPA4* double deficient mouse ES cells and concluded that these genes were dispensable to the ES cell phenotype, since they maintained their ability to self-renew and differentiate similar to wild-type ES lines [14]. However, a recent study by Du et al. [15] demonstrated that siRNA knockdown of *DPPA2* in mouse ES cells resulted in downregulation of marker genes *OCT4* and *NANOG*, accelerated differentiation, and decreased proliferation [15]. In support of these findings, several other knockdown screens have identified these genes as critical in mouse ES cell self-renewal, differentiation, and possible targets of *OCT4* and *SOX2 *[16, 17]. It would be interesting to determine the function of *DPPA2* and *DPPA4* in human stem cells, as these stem cell markers have also been shown to be highly expressed in different types of human cancers [18]. Overall, the data suggests stem cells lines harboring amplifications of *DPPA2* and *DPPA4* may have a selective advantage, and one should be cautious using such lines in their studies. 

The other abnormality acquired in the iPSC line during extended culture was a 285 Kb deletion at 16q23.3 spanning exons 4-5 of the *CDH13* gene (Figure 4(b)). *CDH13*, also known as H-cadherin, has been implicated in cell growth, survival, and proliferation [19]. Downregulation of *CDH13* has been observed in numerous cancer types and has been associated with increased tumor cell aggressiveness [19, 20]. Likewise, overexpression of *CDH13* in cancer cells results in reduced proliferation, increased susceptibility to apoptosis, and a reduction of tumor growth *in vivo *[21, 22]. Moreover, recurrent deletions encompassing *CDH13* have been observed in various cancers including lung cancer, ovarian cancer, and retinoblastoma [20, 23, 24]. This finding is in agreement with Baker et al. [25] who suggest there is a link between cultural adaptation and tumorigenic events that occur *in vivo* [25]. Chromosomal abnormalities such as these found in human stem cells during long-term culture raise obvious concerns about the safety of particular lines.

Although we could provide further examples of the utility of using our updated 135 K StemArray to monitor genomic stability, we believe the example provided here clearly demonstrates the benefits for such testing. Although karyotype analysis is still a popular technique to monitor the genetic integrity of stem cells, many stem cell researchers are beginning to realize the importance of using higher resolution methods to detect submicroscopic alterations. Using an Affymetrix 115,000 single-nucleotide polymorphism (SNP) microarray, Maitra et al. were able to identify an amplification of ~2 Mb on chromosome 8 encompassing the c-MYC oncogene in a high passage ESC line [1].

 Several groups have identified the acquired duplication at 20q11.21 using a wide variety of microarray platforms from low-resolution bacterial artificial chromosome/P1-plasmid artificial chromosome (BAC/PAC) arrays to high-resolution 244,000 probe aCGH arrays [2, 5]. This alteration has been observed in both human ESC and iPSC lines and typically includes the stemness gene DNMT3B. Cell lines containing this duplication tend to grow better, have increased survival, and differentiate slower than wildtype lines. Interestingly, our group and others have also detected amplifications spanning this region which do not contain the DNMT3B gene but do include ID1 [7]. ID1 encodes a helix-loop-helix protein which interacts with the HLH transcription factors, altering their DNA-binding ability [26]. Recently, a study by Martins-Taylor et al. used 135,000 and 385,000 probe microarrays to identify recurrent copy number variations in iPSC lines. Although several small regions commonly acquired in iPSC lines were discovered including 1q31.3, 2p11.2, and 17q21.1, there were no evident candidate genes in these segments with associated functions in stem cells [6].

Although these studies utilizing aCGH technology to characterize stem cell lines were informative, they were all conducted using nontargeted standard catalog microarrays. These microarray platforms are generally designed to tile the entire genome with the resolution dependent on the total number of probes used. Therefore, the majority of stem-cell-related genes in catalog microarrays have little to no coverage, and, as a result, small aberrations spanning these regions are typically missed. In accordance, we routinely detect causative aberrations when testing stem cell samples in our laboratory that had previously appeared normal with karyotype or catalog aCGH microarray analysis. It is important to note that karyotype analysis should not be disregarded completely, because it allows detection of balanced translocations, which is not possible with aCGH. For that reason, we believe both methods should be used in order to obtain a complete genetic profile of a stem cell line. As more data is generated with the 135 K StemArray, we expect to gain new insights into those regions important in stem cell maintenance. In addition, since microarray designs vary wildly in probe placement and gene coverage, it is important for stem cell researchers to agree on specific design parameters to monitor their cell lines if data is to be compared.

## 4. Conclusions

Human stem cell lines that are cultured for an extended period of time are susceptible to chromosomal aberrations. Obtaining a comprehensive genomic profile of these lines is essential, because the acquired structural variations can influence the proliferative ability of the cells. By using a stem-cell-focused microarray such as StemArray, researchers can identify causative aberrations that would otherwise be missed by karyotype analysis and standard catalog arrays. As ES and iPSC lines begin to be used for therapeutic purposes it will be necessary to assess the cells genomic stability with a high-resolution focused array to ensure safety and usefulness.
